# The Efficacy and Safety of Celecoxib in Addition to Standard Cancer Therapy: A Systematic Review and Meta-Analysis of Randomized Controlled Trials

**DOI:** 10.3390/curroncol29090482

**Published:** 2022-08-25

**Authors:** Shi-Yu Ye, Jia-Yi Li, Teng-Hui Li, Yong-Xi Song, Jing-Xu Sun, Xiao-Wan Chen, Jun-Hua Zhao, Yuan Li, Zhong-Hua Wu, Peng Gao, Xuan-Zhang Huang

**Affiliations:** 1 Department of Surgical Oncology and General Surgery, The First Hospital of China Medical University, 155 N. Nanjing Street, Shenyang 110002, China; 2 Key Laboratory of Precision Diagnosis and Treatment of Gastrointestinal Tumors, Ministry of Education, China Medical University, Shenyang 110122, China; 3 Institute of Health Sciences, China Medical University, Shenyang 110122, China

**Keywords:** cancer therapy, meta-analysis, celecoxib, survival, local control

## Abstract

The purpose of this meta-analysis was to evaluate the efficacy and safety of celecoxib, a selective cyclooxygenase-2 (COX-2) inhibitor, in addition to standard anticancer therapy. Randomized controlled trials (RCTs) that evaluated the efficacy and safety of celecoxib-combined cancer therapy were systematically searched in PubMed and Embase databases. The endpoints were overall survival (OS), progression-free survival (PFS), disease-free survival (DFS), objective response rate (ORR), disease control rate (DCR), pathological complete response (pCR), and adverse events (AEs). The results of 30 RCTs containing 9655 patients showed limited benefits in celecoxib-combined cancer therapy. However, celecoxib-combined palliative therapy prolonged PFS in epidermal growth factor receptor (EGFR) wild-type patients (HR = 0.57, 95%CI = 0.35–0.94). Moreover, despite a slight increase in thrombocytopenia (RR = 1.35, 95%CI = 1.08–1.69), there was no increase in other toxicities. Celecoxib combined with adjuvant therapy indicated a better OS (HR = 0.850, 95%CI = 0.725–0.996). Furthermore, celecoxib plus neoadjuvant therapy improved the ORR in standard cancer therapy, especially neoadjuvant therapy (overall: RR = 1.13, 95%CI = 1.03–1.23; neoadjuvant therapy: RR = 1.25, 95%CI = 1.09–1.44), but not pCR. Our study indicated that adding celecoxib to palliative therapy prolongs the PFS of EGFR wild-type patients, with good safety profiles. Celecoxib combined with adjuvant therapy prolongs OS, and celecoxib plus neoadjuvant therapy improves the ORR. Thus, celecoxib-combined cancer therapy may be a promising therapy strategy.

## 1. Introduction

Cancer is a continuing global public health challenge and the second leading cause of death worldwide, closely following cardiovascular (CV) disease [1]. Thus, cancer prevention and therapy are particularly important. Although early diagnosis and therapy strategies of cancer are improving, high mortality and poor prognosis of cancer patients remains a challenge that needs to be addressed [2]. The development of comprehensive therapy strategies may be more beneficial, but the toxicity and side effects of chemotherapy require further consideration. Thus, there remains a need to improve current chemotherapy regimens to maximize the cure rate and minimize chemotherapy-associated toxicity.

Increasing numbers of studies have indicated that tumor occurrence, development, and progression may be promoted by inflammation [3]. The cyclooxygenase-2 (COX-2) enzyme, one of the isoforms of the COX enzyme, catalyzes the production of prostaglandins from arachidonic acid [4]. In addition, COX-2 can be activated by the growth factors, cytokines, and chemokines that are released by the trigger of inflammation [5]. This inflammation-associated metabolic process may play a role in cancer, and COX-2 overexpression is associated with tumor development and progression, tumor characteristics, and poor survival [6,7,8]. Therefore, COX-2 inhibitors are likely to be promising anticancer drugs. Recently, COX-2 inhibitors have been explored in cancer therapy to improve therapeutic efficacy. COX-2 inhibitors may work by antiangiogenic, anti-inflammatory, and proapoptotic mechanisms to promote their antitumor effects [9]. However, there is conflicting data surrounding the efficacy of COX-2 inhibitors combined with anticancer therapy. Furthermore, unlike COX-1 inhibitors, which mainly cause gastrointestinal toxicities due to COX-1 predominating in the gastric mucosa and yielding protective prostaglandins, the COX-2 inhibitors may lead to increased CV thrombotic events by decreasing vasodilatory and antiaggregatory vascular prostacyclin (PGI_2_) production, which results in the increase of prothrombotic eicosanoids (e.g., thromboxane A2) [10,11]. Therefore, it remains unclear whether the additional COX-2 inhibitors in tumor therapy are clinically beneficial and safe for cancer patients. 

Celecoxib, a highly selective COX-2 inhibitor, is an established anti-inflammatory drug that has been used for treating osteoarthritis, rheumatoid arthritis, familial adenomatous polyposis, and acute pain for many years [12]. Numerous studies have reported the chemopreventive efficacy of celecoxib in several precancerous lesions and cancer types, including colon polyp, colorectal adenomas, lung cancer, and prostate cancer [13,14,15,16,17]. Furthermore, celecoxib was found to prevent tumor growth through multiple pathways and targets [18,19,20,21,22,23,24,25,26]. In terms of the molecular mechanism of celecoxib, it can regulate FAK, Cx43, p21, and Ki-67 molecules, and block AKT activation [20,22,24], which can inhibit tumor cell proliferation and induce apoptosis. Moreover, celecoxib is involved in ERK1/2 MAPK and PI3K/AKT pathways, exerting an antiangiogenic effect [18,23]. Celecoxib is also involved in COX-2/PGE_2_/EP_2_/p-AKT/p-ERK and PGE_2_/NF-kB pathways, which prevents the invasion and metastasis of tumor cells [25,26]. Previous animal studies and clinical studies have reported that the administration of celecoxib with chemotherapy could significantly relieve chemotherapy-related toxicities [27,28]. In terms of celecoxib in addition to cancer therapy, some clinical studies reported that additional celecoxib in palliative hormone therapy seemingly contributes to reverse endocrine resistance in breast cancer patients and improves the clinical efficacy of preoperative chemoradiation in rectal cancer [29,30], but no significant clinical benefit was found in celecoxib-combined palliative therapy for lung cancer and colorectal cancer, nor in celecoxib-combined adjuvant hormone therapy for breast cancer [31,32,33]. Since different cancers have varying biological characteristics and exhibit different responses to therapy regimens, the conflicting data may be attributed to cancer type and therapy approach. It is therefore critical to explore whether celecoxib is beneficial to patient prognosis and, if so, which potential patient populations will benefit from celecoxib–targeted anticancer therapy.

In the present study, we collected relevant RCTs and conducted a systematic meta-analysis to assess the efficacy and safety of celecoxib combined with standard anticancer therapy in various cancers and therapy strategies.

## 2. Materials and Methods

### 2.1. Literature Search

We systematically searched PubMed and Embase databases up to November 2021 for all relevant RCT studies that evaluated the efficacy and safety of celecoxib in addition to standard therapy for cancer patients, including palliative chemotherapy, adjuvant chemotherapy, and neoadjuvant chemotherapy. The search strategy used the main keywords and MeSH terms of “celecoxib”, “Celebrex”, “cancer”, “tumor”, “neoplasm”, “carcinoma”, “palliative”, “postoperative”, “post-operative”, “adjuvant”, “neoadjuvant”, “preoperative”, “pre-operative”, “chemotherapy”, “chemoradiation”, “hormone therapy”, “randomized trial”, “randomised trial”, “randomized study”, and “randomised study”. In addition, the reference lists of relevant literature were manually screened to identify any potentially eligible literature.

### 2.2. Eligibility Criteria

This meta-analysis aimed to evaluate whether additional celecoxib in standard therapy improved the prognosis for cancer patients. The inclusion criteria were as follows: (1) Patients: patients were pathologically diagnosed with cancer and treated with standard anticancer therapy. (2) Intervention: cancer patients received standard therapy with celecoxib as opposed to standard therapy alone or with placebo. (3) Comparison: the control group received standard therapy with or without placebo. (4) Outcomes: the study outcomes consisted of overall survival (OS), progression-free survival (PFS), disease-free survival (DFS), objective response rate (ORR), disease control rate (DCR), pathological complete response (pCR), and drug safety; the outcome measures and corresponding 95% confidence intervals (CIs) were reported. (5) Study design: the studies were RCTs. Given that radiotherapy is the local therapy for cancer, and we were focusing on assessing the systemic effects of celecoxib in cancer therapy, we excluded celecoxib-combined radiotherapy studies. If several studies were based on the same patient population, the relevant data of interest were merged into the most informative study, which was enrolled.

### 2.3. Data Extraction and Assessment of Study Quality

The relevant data of eligible RCTs extracted by two reviewers independently were as follows: first author, country, year of publication, therapy type, cancer type, sample size, age, follow-up period, long-term efficacy, short-term efficacy, and drug safety. The two reviewers resolved all discrepancies through comprehensive discussion in accordance with the PRISMA statement [34]. The study quality was evaluated on the basis of the Jadad criteria [35].

### 2.4. Statistical Analysis

The primary endpoints were long-term efficacies, including OS, PFS, and DFS. The secondary endpoints were short-term efficacies (ORR, DCR, and pCR) and adverse events (AEs). We extracted the hazard ratios (HRs), relative risks (RRs), and 95%CIs from each trial. In studies that did not obtain direct outcomes, HRs and 95%CIs were calculated according to the method designed by Tierney [36], and the ORR and DCR were calculated from the complete response (CR), partial response (PR), stable disease (SD), and progressive disease (PD) data according to the Response Evaluation Criteria in Solid Tumors (RECIST) guidelines [37].

The Cochran Q test and I^2^ index were conducted to evaluate the heterogeneity among RCTs, with I^2^ < 50% and *p* > 0.1 indicating no significant heterogeneity [38]. When significant heterogeneity was found, a random-effects model was conducted, otherwise a fixed-effects model was conducted. Overall analyses across all therapy approaches were made based on all the enrolled studies. Then, we focused on analyzing the efficacy and safety profile of celecoxib in addition to anticancer therapy, such as palliative, adjuvant, and neoadjuvant therapies. Simultaneously, subgroup analyses on different therapy methods were performed based on therapy type (chemo/chemoradiation/hormone therapy), cancer type, COX-2 status, the urinary metabolite of prostaglandin E_2_ (PGEM) status, epidermal growth factor receptor (EGFR) status, use of nonsteroidal anti-inflammatory drugs (NSAIDs), World Health Organization (WHO) performance score, and sample size. Begg’s and Egger’s tests were used to evaluate the publication bias [39,40].

STATA version 12.0 (Stata Corporation, College Station, TX, USA) was used to perform all statistical analyses. When a two-sided *p*-value was < 0.05, the results were regarded as statistically significant. The protocol of this systematic review was not a routine local research protocol on the PROSPERO registry by the time of completion of the work. Therefore, though the comprehensive web search showed no similar registered study in ROSPERO, we do not have a registration number.

## 3. Results

### 3.1. Study Selection and Associated Characteristics

**Figure 1:**
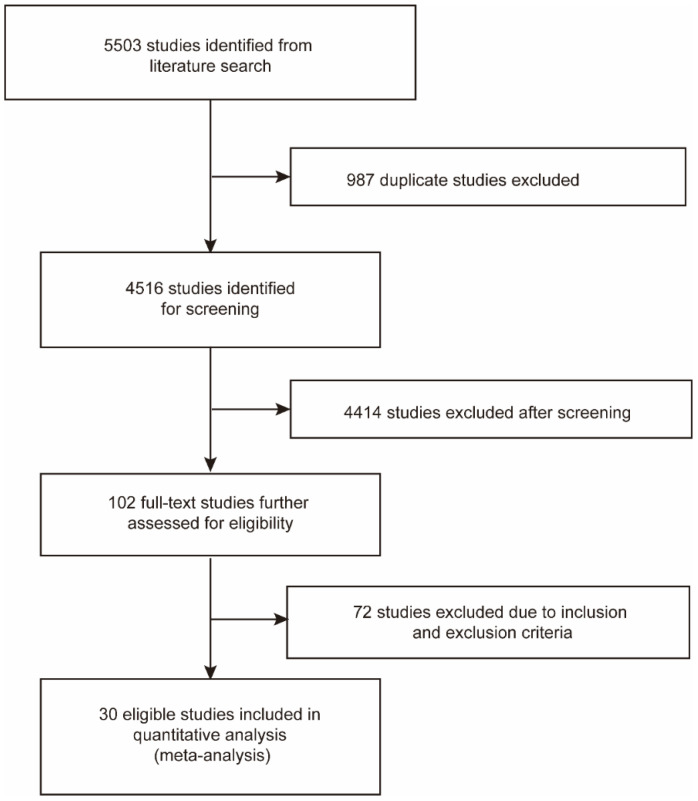
Flow chart of the literature search and study selection process.

A total of 1368 and 4135 relevant studies were identified from PubMed and Embase databases, respectively. Of these, 987 duplicate studies were excluded. After screening the remaining 4516 studies according to the title and abstract, we excluded 4414 studies on the basis of eligibility criteria. Then we reviewed the full texts of the selected 102 studies and excluded 72 studies due to irrelevant data. Finally, 30 studies were included in this meta-analysis (Figure 1) [29,30,31,32,33,41,42,43,44,45,46,47,48,49,50,51,52,53,54,55,56,57,58,59,60,61,62,63,64,65].

The 30 enrolled studies were published from 2004 to 2021 and contained a total of 9655 patients. Of these studies, Edelman et al. (2017), Koch et al. (2011), and Hamy et al. (2019) had merged the relevant data from Edelman et al. (2014), Gulyas et al. (2018), and Pierga et al. (2010) based on the same patient population, respectively [66,67,68]. Among the included studies, celecoxib was combined with palliative therapy in 20 studies, with adjuvant therapy in three studies and neoadjuvant therapy in seven. The enrolled studies were related to various cancers, including nine breast cancer studies, eight lung cancer studies, six colorectal cancer studies, two gynecological cancer studies, two head and neck cancer studies, one gastric cancer study, one prostate cancer study, and one bladder cancer study. The main characteristics and quality assessments of each included study are shown in Table S1.

### 3.2. Overall Efficacy of Celecoxib in Standard Anticancer Therapy

In a total of 30 enrolled studies, 17 reported OS, and the pooled result showed no improvement in celecoxib-combined cancer therapy (HR = 1.00, 95%CI = 0.92–1.08). Moreover, the addition of celecoxib in cancer therapy did not improve PFS (HR = 1.02, 95%CI = 0.91–1.13), nor DFS (HR = 1.05, 95%CI = 0.80–1.38). As for the local control, analyses indicated that patients in the celecoxib-combined cancer therapy group obtained a better ORR than the control group (39.3% versus 31.8%; RR = 1.13, 95%CI = 1.03–1.23), but not DCR (71.9% versus 68.9%; RR=1.05, 95%CI = 0.99–1.11). Furthermore, no improvement in pCR was found in the patients who received celecoxib-combined cancer therapy (RR = 1.28, 95%CI = 0.88–1.85). These analysis results are shown in Table 1.

### 3.3. Celecoxib and Overall Survival (OS) in Palliative Therapy

**Figure 2:**
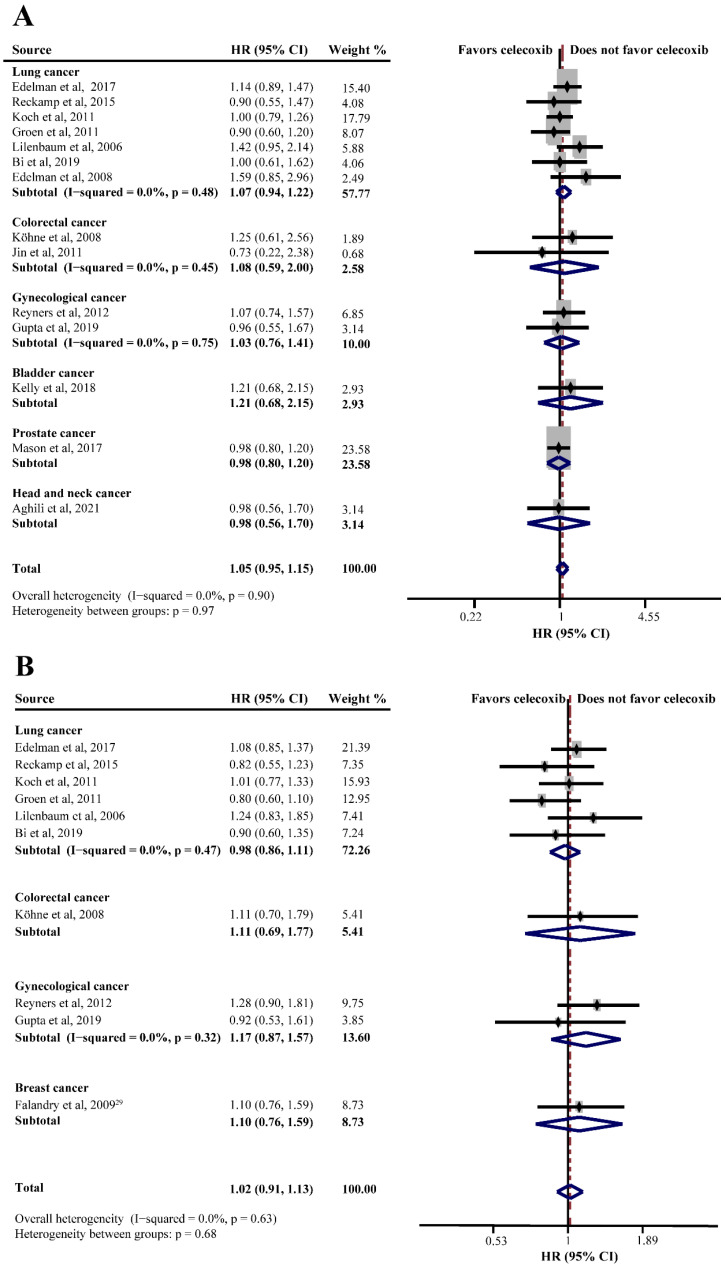
Efficacy of celecoxib-combined palliative therapy in different cancer types on overall survival (OS) (**A**) and progression-free survival (PFS) (**B**). HR: hazard ratio; CI: confidence interval [29,31,41,42,48,50,51,52,56,58,59,60,61,62,63].

Twenty RCTs consisting of 4688 patients assessed the efficacy of celecoxib in addition to standard palliative therapy. Of these, 14 studies reported the OS of celecoxib-combined therapy versus standard therapy alone or with placebo. No improvement was shown in the OS (HR = 1.05, 95%CI = 0.95–1.15), without significant heterogeneity (I^2^ = 0.0%) (Figure 2A). Subgroup analysis based on different cancers showed no efficacy of celecoxib combined with palliative therapy on OS (lung cancer: HR = 1.07, 95%CI = 0.94–1.22; colorectal cancer: HR = 1.08, 95%CI = 0.59–2.00; gynecological cancer: HR = 1.03, 95%CI = 0.76–1.41) (Figure 2A). Moreover, the analyses revealed that celecoxib-combined chemotherapy or chemoradiotherapy did not prolong OS (chemotherapy: HR = 1.08, 95%CI = 0.96–1.21; chemoradiotherapy: HR = 0.99, 95%CI = 0.69–1.43). The addition of celecoxib to first-line palliative therapy did not obtain a better efficacy (HR = 1.03, 95%CI = 0.93–1.15), nor to ≥first-line palliative therapy (HR = 0.93, 95%CI = 0.64–1.34). Moreover, our meta-analyses revealed no beneficial effects with respect to COX-2 status, PGEM status, EGFR status, use of NSAIDs, and WHO performance status. Furthermore, we conducted a subgroup analysis after stratifying by sample size, and the results were still insignificant. The detailed results of the subgroup analysis for the OS of celecoxib-combined palliative therapy are shown in Table 2.

**Figure 3:**
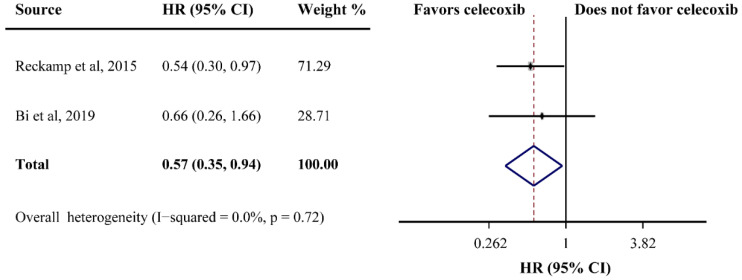
Efficacy of celecoxib-combined palliative therapy on progression-free survival (PFS) for EGFR wild-type status patients. HR: hazard ratio; CI: confidence interval [48,59].

PFS was reported in ten studies and the pooled HR did not indicate a beneficial effect from celecoxib combined with palliative therapy (HR = 1.02, 95%CI = 0.91–1.13), without obvious heterogeneity (I^2^ = 0.0%) (Figure 2B). Subgroup analyses according to cancer type revealed that celecoxib-combined palliative therapy did not prolong PFS (lung cancer: HR = 0.98, 95%CI = 0.86–1.11; gynecological cancer: HR = 1.17, 95%CI = 0.87–1.57) (Figure 2B). Moreover, celecoxib combined with chemotherapy made no improvement in PFS (HR = 1.02, 95%CI = 0.91–1.15). In addition, celecoxib did not improve PFS when added to first- and ≥ first-line palliative therapies. Likewise, the PFS based on COX-2 status and PGEM status did not significantly differ (high COX-2 status: HR = 1.03, 95%CI = 0.82–1.30; high PGEM status: HR = 0.71, 95%CI = 0.48–1.07; low PGEM status: HR = 1.05, 95%CI = 0.78–1.42). Interestingly, subgroup analysis of EGFR status revealed that EGFR wild-type status was beneficial to PFS (HR = 0.57, 95%CI = 0.35–0.94) (Figure 3). The subgroup analysis results for the PFS of celecoxib-combined palliative therapy are shown in Table 2.

### 3.4. Celecoxib and Local Control in Palliative Therapy

In the celecoxib-combined group versus the control group, the ORR was 33.5% versus 27.2%, and the DCR was 71.9% versus 68.9%, respectively. The pooled RRs showed that the addition of celecoxib to palliative therapy made no improvement in the ORR or DCR for patients receiving palliative therapy (ORR:RR = 1.08, 95%CI = 0.96–1.21; DCR:RR = 1.05, 95%CI = 0.99–1.11) (Figure S1A,B). In addition, subgroup analyses based on different cancer types revealed that celecoxib was not beneficial to the local control, no matter which type of cancer (Figure S1A,B). Our subgroup analysis revealed that the local control was not improved when celecoxib was combined with palliative chemotherapy or palliative hormone therapy. Furthermore, analyses according to therapy line revealed similar results. The subgroup analysis results for the local control efficacy of celecoxib combined with palliative therapy are shown in Table S2.

### 3.5. Celecoxib and Adjuvant Therapy

**Figure 4:**
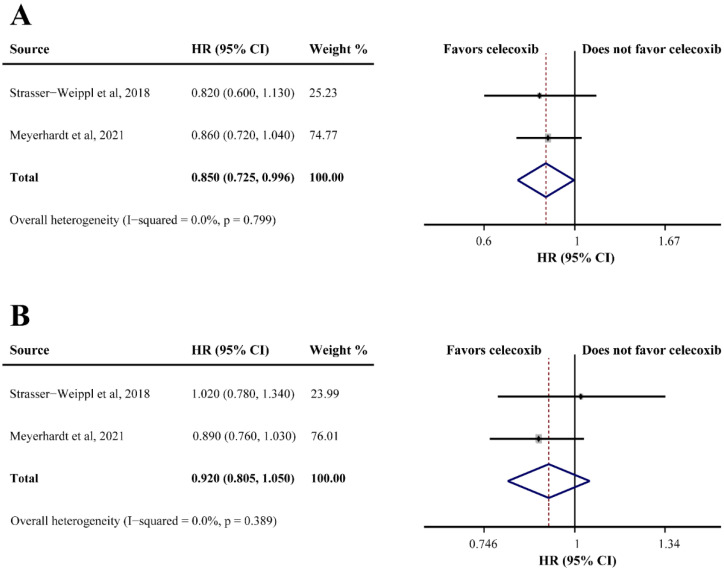
Efficacy of celecoxib-combined adjuvant therapy on overall survival (OS) (**A**) and disease-free survival (DFS) (**B**). HR: hazard ratio; CI: confidence interval [32,47].

Two studies reported the OS of patients receiving celecoxib-combined adjuvant therapy, and the pooled HR indicated that the addition of celecoxib produced a better OS (HR = 0.850, 95%CI = 0.725–0.996) (Figure 4A). However, celecoxib was found ineffective for DFS (HR = 0.920, 95%CI = 0.805–1.050) (Figure 4B). Non-significant heterogeneities were found in the analyses described above (OS: I^2^ = 0.0%; DFS: I^2^ = 0.0%) (Figure 4A,B). One study reported that celecoxib alone was used as maintenance therapy after the completion of adjuvant therapy versus observation without celecoxib in breast cancer, and a significantly improved DFS was observed in the celecoxib group (66% and 41.9%, respectively; HR = 0.17, 95%CI = 0.07–0.40).

### 3.6. Celecoxib and Neoadjuvant Therapy

**Figure 5:**
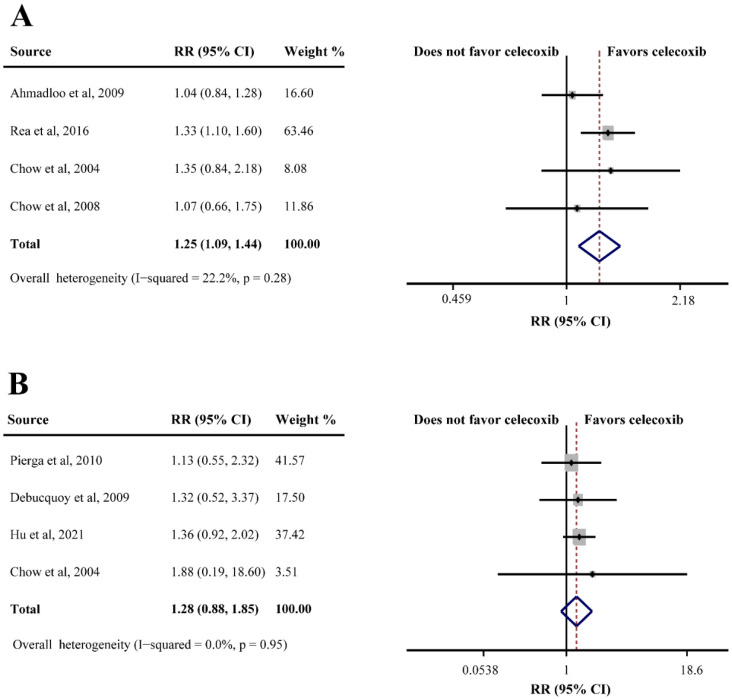
Efficacy of celecoxib-combined neoadjuvant therapy on objective response rate (ORR) (**A**) and pathological complete response (pCR) (**B**). RR: relative risk; CI: confidence interval [30,43,45,53,54,55,68].

Seven studies consisting of 735 patients evaluated the efficacy of celecoxib plus neoadjuvant therapy (OS, DFS, ORR, and pCR). Only one study reported OS in breast cancer (HR = 1.71, 95%CI = 0.88–3.33), which indicated that celecoxib-combined neoadjuvant therapy had no effect on OS. However, the same study showed that celecoxib combined with neoadjuvant therapy had relative to poor DFS (HR = 1.70, 95%CI = 1.00–2.88). In addition, four studies reporting ORR data found that celecoxib had a beneficial effect when combined with neoadjuvant therapy (75.9% in the celecoxib group compared to 62.5% in the control group; RR = 1.25, 95%CI = 1.09–1.44) (Figure 5A). However, an analysis of four studies based on pCR showed that celecoxib had no significant efficacy in neoadjuvant therapy (RR = 1.28, 95%CI = 0.88–1.85), without substantial heterogeneity (I^2^ = 0.0%) (Figure 5B).

### 3.7. Safety of Celecoxib

Drug safety is an important issue to be taken into consideration for clinical decision-making. Thus, the safety of celecoxib was estimated by analyzing the commonly reported grade ≥ 3 toxicities. Our analysis indicated that celecoxib-combined palliative therapy only increased the incidence of thrombocytopenia (RR = 1.35, 95%CI = 1.08–1.69), and no differences in other common hematological toxicities were observed. Furthermore, our data showed no increase in gastrointestinal toxicities when celecoxib was added to anticancer therapy (overall gastrointestinal toxicities: RR = 1.19, 95%CI = 0.94–1.52), including heartburn/dyspepsia, diarrhea, nausea/vomiting, and constipation. Though celecoxib has been reported to cause CV events, the results in our analyses showed no difference between the celecoxib-combined and control groups, including cardiac ischaemia/infarction, cerebrovascular ischaemia, and thrombosis/thrombus/embolism. Moreover, no increased risk in other common toxicities such as circulatory toxicities, musculoskeletal toxicities, neurotoxicity, fatigue, anorexia, rash, mucositis, infection, and pain was observed after celecoxib therapy (Table S3).

For celecoxib-combined adjuvant therapy, the occurrence rates of grade ≥ 3 AEs for the celecoxib and control groups were 51.7% and 50.8%, respectively Furthermore, for celecoxib-combined neoadjuvant therapy, three RCTs reported no obvious differences in toxicities between the celecoxib and control groups except for skin reaction and increased aspartate aminotransferase levels. 

### 3.8. Publication Bias

Begg’s and Egger’s tests showed no significant publication bias in our meta-analysis (Figures S2–S5, Table S4).

## 4. Discussion

Cancer remains a life-threatening disease and the second leading cause of death worldwide [69]. As early as 46 years ago, chemoprevention was introduced as an approach to prevent cancer occurrence [70], and researchers gradually discovered that some medicines have additional effects of cancer prevention in the precancer period, including the precancerous lesions [71,72]. Limitations in current anticancer therapeutic strategies highlight the importance of developing more efficient regimens to improve prognosis. As inflammation is closely connected to the development of cancer, COX-2, a proinflammatory enzyme, may be a novel target to improve the therapeutic effect. Recent studies have demonstrated that COX-2 plays an essential role in cancer initiation, progression, invasion, and metastasis [73]. Overexpressed COX-2 was found in several cancer types and promotes the malignant biological behavior of tumor cells though various cytokines and signature pathways [5,8]. COX-2 inhibitors have been found to have cancer prevention and anticancer activities [71,74]. Among them, rofecoxib has been found to suppress the growth of tumor cells [75]. Similarly, valdecoxib exhibited potent growth inhibition and cytotoxic effects against cancer cells [75]. A study has reported that apricoxib plus erlotinib improved the survival of non-small cell lung cancer (NSCLC) patients younger than 65 years old [76]. As a widespread COX-2 inhibitor, celecoxib has cancer preventive effects across primary, secondary, and tertiary prevention [77]. For primary chemoprevention, celecoxib can effectively prevent the occurrence of lung cancer in former smokers [17]. As to second chemoprevention, celecoxib is an effective agent for the prevention of colorectal adenomas and familial adenomatous polyposis [14,77]. In terms of tertiary chemoprevention, also known as the clinical therapy phase, clinical studies reported that celecoxib improved the clinical efficacy of breast cancer, gastric cancer, rectal cancer, colorectal cancer, and head and neck cancer [29,30,46,50,56]. Celecoxib may exert its anticancer effects by reducing the production of COX-2-dependent PGE_2_ and then inhibiting the COX-2/PGE_2_/EP_2_/p-AKT/p-ERK and PGE_2_/NF-kB pathways [25,26]. However, the evidence remains conflicting in the clinical effects of celecoxib-combined therapy. Here, we aimed to assess the efficacy and safety profile of celecoxib in addition to anticancer therapy, such as palliative, adjuvant, and neoadjuvant therapies. Unlike the previous studies that used the epidemiological information of medication use to explore the prognosis, this study is the first meta-analysis to include RCTs that compared celecoxib-combined therapy and placebo-combined therapy or therapy alone. As far as we know, this is also the first meta-analysis to evaluate the efficacy and safety of celecoxib combined with standard cancer therapy based on different cancer types, concomitant therapy strategies, and therapy stages.

The current meta-analysis consisted of 30 RCTs and included data from 9655 cancer patients. Results showed that there were limited benefits in celecoxib-combined cancer therapy for OS, PFS, DFS, DCR, and pCR, but there was a better ORR (RR = 1.13, 95%CI = 1.03–1.23). Although celecoxib combined with palliative therapy showed no improvement in patient survival and the local control of the tumor, EGFR wild-type patients had a prolonged PFS with celecoxib-combined therapy (HR = 0.57, 95%CI = 0.35–0.94). In addition, celecoxib produced no increase in hematological, gastrointestinal, or other AEs, except for a slight increase in thrombocytopenia (RR = 1.35, 95%CI = 1.08–1.69). In celecoxib-combined adjuvant therapy studies, a better OS was reported in the celecoxib group compared to the control group (HR = 0.850, 95%CI = 0.725–0.996). Interestingly, one study reported that celecoxib alone administered as maintenance therapy to patients with non-metastatic triple-negative breast cancer after adjuvant therapy could prolong DFS (HR = 0.17, 95%CI = 0.07–0.40) [64]. Furthermore, celecoxib improved the ORR for patients receiving celecoxib-combined neoadjuvant therapy for breast cancer (RR = 1.25, 95%CI = 1.09–1.44).

Our meta-analysis indicated limited benefits of OS, PFS, DFS, DCR, and pCR, but a better ORR in celecoxib-combined cancer therapy. Based on the more detailed analyses, there were no survival and local control benefits associated with celecoxib-combined palliative therapy. This insignificant result may be because patients receiving palliative therapy usually had advanced or metastatic cancer, and thus the shorter survival contributed to insufficient intake of celecoxib and a subsequent reduction in its potential anticancer effects [78]. Unlike COX-1 inhibitors which cause gastrointestinal toxicities, the COX-2 inhibitors mainly cause CV toxicities, and the potential risk for CV events needs to be considered during clinical decision-making. Previous studies have reported that celecoxib has the preventive effect of colorectal adenomas, but it cannot be routinely recommended for this indication due to the increased risk of CV events [14]. Fortunately, based on the analyses in this study, there was no significant increase in CV events including cardiac ischaemia/infarction, cerebrovascular ischaemia, and thrombosis/thrombus/embolism. Moreover, in the five studies used to analyze CV toxicity, there was only one that stopped because of a small increased risk of CV events [42]. There is still a need for further studies involving large and representative samples to confirm the celecoxib safety in combined cancer therapy due to the limited data. Further, the pooled results showed no difference between the toxicities of celecoxib and control groups, except for a slightly higher occurrence of thrombocytopenia when celecoxib was combined with palliative therapy. Since high COX-2 expression has been found in bone marrow stromal cells in cancer patients, that the addition of a COX-2 inhibitor delays the recovery of hematopoietic progenitor cells after chemotherapy may be the cause of thrombocytopenia [79,80]. Our findings showed that celecoxib prolonged PFS in patients with EGFR wild-type NSCLC, suggesting that celecoxib-combined palliative therapy is a promising therapy strategy for this population. The EGFR-activated-type patients with advanced NSCLC were reported to benefit from the EGFR tyrosine kinase inhibitor (TKI) therapy [81,82], but the EGFR wild-type patients had no effective targeted therapies other than conventional chemotherapy. Therefore, EGFR may be a potential biomarker to identify the efficacy of celecoxib-combined therapy. 

Moreover, though the high level of prostaglandin E_2_ (PGE_2_), induced by the overexpressed COX-2, can cause the proliferation of tumor cells, stimulate Bcl-2 protein expression that inhibits apoptosis, and promote the malignant biological behavior of tumors [83], several studies have revealed that COX-2 inhibitors may exert anticarcinogenic effects, not only through COX-2-dependent but also COX-2-independent mechanisms. Moreover, the synthesis of COX-2-dependent PGEM was not the main cause of COX-2 inhibitor efficacy [74]. These findings may explain why celecoxib could not improve the survival and local control of the tumor, independent of COX-2 status or PGEM status. However, further studies are needed to confirm the relationship between celecoxib-combined cancer therapy and the status of COX-2 and PGEM, due to the limited data from the relevant studies. Moreover, early use of celecoxib may be beneficial, since a better OS was observed in celecoxib-combined adjuvant therapy, and a better ORR was reported in celecoxib-combined neoadjuvant therapy. Clinicians should carefully weigh the clinical benefits against the potential increase in CV events during clinical decision-making. In addition, the better ORR in neoadjuvant therapy was obtained by pooling data from breast cancer studies, as breast cancer generally has high long-term survival rates. Furthermore, the development of bone marrow micro-metastasis and subsequent osteolytic bone metastases in breast cancer patients may be due to the overexpression of COX-2 in primary breast cancer cells. Since studies have shown that the COX-2 inhibitor, celecoxib, inhibited this metastatic process [84], these findings may explain why the celecoxib-combined neoadjuvant therapy is beneficial for breast cancer patients.

Our meta-analysis study has many strengths. Firstly, the present study included all relevant RCTs, and therefore the heterogeneities were found to be low. Secondly, we systematically evaluated all kinds of tumors, and we enhanced the stability and reliability of our results and conclusions by an in-depth subgroup analysis based on different therapy stages, cancer types, and more detailed subgroups. Thirdly, the addition of celecoxib to adjuvant therapy prolonged OS. 

The present study also had several limitations. Firstly, subgroup analyses were not performed on detailed therapeutic regimens and celecoxib dosage due to limited data, which may influence the evaluation of the efficacy of celecoxib. Secondly, we made the subgroup according to cancer molecular type, but the limited data from enrolled studies only supported the analyses based on COX-2, EGFR, and PEGM status, and we found EGFR wild-type patients obtain a prolonged PFS in celecoxib-combined palliative therapy. Therefore, further studies are needed to explore the efficacy of celecoxib in cancer patients with other molecular types based on different cancers. Thirdly, we were unable to obtain detailed individual data since this study was based on previously published data from the enrolled RCTs. Thus, we could not perform an in-depth subgroup analysis according to prognosis-related factors such as age, sex, ethnic group, and TNM stage. In addition, the efficacy of celecoxib may have been underestimated since we could not completely control these inherently confounding factors in the included studies. 

## 5. Conclusions

The addition of celecoxib to palliative therapy cannot improve survival and local control rates except for the PFS of EGFR wild-type patients, without obvious toxicities and AEs. In terms of adjuvant therapy, the addition of celecoxib can prolong the OS but not the DFS. Moreover, the combination of celecoxib with neoadjuvant therapy can improve the ORR for breast cancer. In general, further studies evaluating the celecoxib efficacy on cancer therapy should be conducted with caution in certain populations to reduce high clinical trial costs and use of medical recourses.

## Figures and Tables

**Table 1:** The analysis results across all therapy approaches of celecoxib combined with cancer therapy for cancer patients.

	Index of Risk	95%CI	*p*-Value	Heterogeneity (I^2^, *p*-Value)
Overall survival (OS)	HR = 1.00	0.92–1.08	0.57	0.00%, 0.93
Progression-free survival (PFS)	HR = 1.02	0.91–1.13	0.76	0.00%, 0.63
Disease-free survival (DFS)	HR = 1.05	0.80–1.38	0.06	64.40%, 0.73
Objective response rate (ORR)	RR = 1.13	1.03–1.23	0.33	10.20%, 0.01
Disease control rate (DCR)	RR = 1.05	0.99–1.11	0.11	0.00%, 0.76
Pathological complete response (pCR)	RR = 1.28	0.88–1.85	0.53	0.00%, 0.95

HR: hazard ratio; RR: relative risk; CI: confidence interval; I^2^: showed the degree of heterogeneity.

**Table 2:** The results of subgroup analyses on the survival efficacy of celecoxib combined with palliative therapy.

	HR	95%CI	*p*-Value	Heterogeneity (I^2^, *p*-Value)
***Overall survival***				
**Concomitant therapy methods**				
Chemotherapy	1.08	0.96–1.21	0.23	0.00%, 0.79
Chemoradiotherapy	0.99	0.69–1.43	0.96	0.00%, 0.96
**Therapy stages**				
First-line	1.03	0.93–1.15	0.59	0.00%, 0.91
≥First-line	0.93	0.64–1.34	0.68	0.00%, 0.86
**COX-2 status**				
High COX-2 status	0.94	0.67–1.31	0.70	62.60%,0.05
Low COX-2 status	1.13	0.78–1.64	0.53	51.50%, 0.13
**PGEM status**				
High PGEM status	0.79	0.47–1.34	0.39	0.00%, 0.49
Low PGEM status	1.27	0.89–1.81	0.19	0.00%, 0.90
**EGFR status**				
EGFR wild-type	1.03	0.62–1.70	0.92	0.00%, 0.99
**Use of NSAIDs**				
No use of NSAIDs	0.98	0.81–1.18	0.80	0.00%, 0.61
Use of NSAIDs	0.66	0.23–1.91	0.44	64.30%, 0.09
**Performance Status (PS, WHO)**				
PS: 0	0.88	0.66–1.19	0.41	0.00%, 0.89
PS: ≥1	1.02	0.83–1.25	0.86	0.00%, 0.40
**Sample size**				
<200	1.12	0.92–1.36	0.27	0.00%, 0.71
≥200	1.02	0.91–1.15	0.69	0.00%, 0.87
***Progression-free survival***				
**Concomitant therapy strategies**				
Chemotherapy	1.02	0.91–1.15	0.75	0.00%, 0.47
**Therapy stages**				
First-line	1.01	0.89–1.15	0.83	0.00%, 0.44
≥First-line	0.85	0.62–1.18	0.34	0.00%, 0.74
**COX** **-2** **status**				
High COX-2 status	1.03	0.82–1.30	0.79	0.00%, 0.86
**PGEM status**				
High PGEM status	0.71	0.48–1.07	0.10	0.00%, 0.74
Low PGEM status	1.05	0.78–1.42	0.73	0.00%, 0.33
**EGFR status**				
EGFR wild-type	0.57	0.35–0.94	0.03	0.00%, 0.72
**Sample size**				
<200	0.99	0.81–1.20	0.90	0.00%, 0.63
≥200	1.03	0.90–1.18	0.65	9.70%, 0.35

HR: hazard ratio; CI: confidence interval; I^2^: showed the degree of heterogeneity; COX-2: cyclooxygenase-2; PGEM: the urinary metabolite of prostaglandin E_2_; EGFR: epidermal growth factor receptor; NSAIDs: nonsteroidal anti-inflammatory drugs; PS: performance status; WHO: World Health Organization.3.4. Celecoxib and Progression-Free Survival (PFS) in Palliative Therapy.

## Supplementary Materials

The following supporting information can be downloaded at: https://www.mdpi.com/article/10.3390/curroncol29090482/s1. Figure S1: local control efficacy of celecoxib-combined palliative therapy in different cancer types on objective response rate (ORR) (A) and disease control rate (DCR) (B); RR: relative risk; CI: confidence interval. Figure S2: Begg’s funnel plot of publication bias analysis in celecoxib-combined standard cancer therapy on overall survival (OS) (A), progression-free survival (PFS) (B), disease-free survival (DFS) (C), objective response rate (ORR) (D), disease control rate (DCR) (E), and pathological complete response (pCR) (F). Figure S3: Begg’s funnel plot of publication bias analysis in celecoxib-combined palliative therapy on overall survival (OS) (A), progression-free survival (PFS) (B), objective response rate (ORR) (C), and disease control rate (DCR) (D). Figure S4: Begg’s funnel plot of publication bias analysis in celecoxib-combined adjuvant therapy on overall survival (OS) (A) and disease-free survival (DFS) (B). Figure S5: Begg’s funnel plot of publication bias analysis in celecoxib-combined neoadjuvant therapy on objective response rate (ORR) (A) and pathological complete response (pCR) (B). Table S1: the baseline patient characteristics of the included RCTs. Table S2: the subgroup analysis results for the local control efficacy of celecoxib-combined palliative therapy. Table S3: the analysis results of toxicities in celecoxib combined with palliative therapy for cancer patients. Table S4: the analysis results of publication bias in celecoxib combined with standard cancer therapy.

## Abbreviations

COX-2: cyclooxygenase-2; RCTs: randomized controlled trials; OS: overall survival; PFS: progression-free survival; DFS: disease-free survival; ORR: objective response rate; DCR: disease control rate; pCR: pathological complete response; AEs: adverse events; EGFR: epidermal growth factor receptor; CIs: confidence intervals; HRs: hazard ratios; RRs: the relative risks; CR: complete response; PR: partial response; SD: stable disease; PD: progressive disease; CV: cardiovascular; RECIST: Response Evaluation Criteria in Solid Tumors; PGEM: the urinary metabolite of prostaglandin E_2_; NSAIDs: nonsteroidal anti-inflammatory drugs; WHO: World Health Organization; NSCLC: non-small cell lung cancer; TKI: tyrosine kinase inhibitor.
