# Supplementary material for: The Efficacy and Safety of Celecoxib in Addition to Standard Cancer Therapy: A Systematic Review and Meta-Analysis of Randomized Controlled Trials
Source: Curr Oncol. 2022 Aug 25;29(9):6137–53. doi: 10.3390/curroncol29090482 (PMC9497539; doi:10.3390/curroncol29090482)
Supplement: Supplementary file 1 [file curroncol-29-00482-s001.zip › curroncol-1811767-supplementary.pdf]

The number of supplemental figures and tables: 9

Figure S1: Local control efficacy of celecoxib-combined palliative therapy in different cancer types on objective response rate (ORR) (A) and disease control rate (DCR) (B). RR: relative risk; CI: confidence interval.

Figure S2: Begg's funnel plot of publication bias analysis in celecoxib-combined standard cancer therapy on overall survival (OS) (A), progression-free survival (PFS) (B), disease-free survival (DFS) (C), objective response rate (ORR) (D), disease control rate (DCR) (E), and pathological complete response (pCR) (F).

Figure S3: Begg's funnel plot of publication bias analysis in celecoxib-combined palliative therapy on overall survival (OS) (A), progression-free survival (PFS) (B), objective response rate (ORR) (C) and disease control rate (DCR) (D).

Figure S4: Begg's funnel plot of publication bias analysis in celecoxib-combined adjuvant therapy on overall survival (OS) (A) and disease-free survival (DFS) (B).

Figure S5: Begg's funnel plot of publication bias analysis in celecoxib-combined neoadjuvant therapy on objective response rate (ORR) (A) and pathological complete response (pCR) (B).

Table S1: The baseline patient characteristics of the included RCTs.

Table S2: The subgroup analysis results for the local control efficacy of celecoxib-combined palliative therapy.

Table S3: The analysis results of toxicities in celecoxib combined with palliative therapy for cancer patients.

Table S4: The analysis results of publication bias in celecoxib combined with standard cancer therapy.

PRISMA checklist

A

Source	RR (95% CI)	Celecoxib	Control	Weight %
**Lung cancer**				
Reckamp et al, 2015	0.69 (0.37, 1.30)	12/53	17/52	5.18
Koch et al, 2011	1.16 (0.85, 1.59)	57/158	49/158	14.78
Groen et al, 2011	1.00 (0.81, 1.24)	104/290	109/305	32.06
Lilenbaum et al, 2006	0.49 (0.09, 2.56)	2/51	4/50	1.22
Edelman et al, 2008	1.20 (0.66, 2.19)	27/90	11/44	4.46
Zhou et al, 2007	1.26 (0.60, 2.63)	11/32	9/33	2.67
**Subtotal (I−squared = 0.0%, p = 0.63)**	**1.03 (0.88, 1.21)**	**213/674**	**199/642**	**60.37**
**Colorectal cancer**				
Maiello et al, 2006	0.80 (0.46, 1.39)	14/39	17/38	5.20
Köhne et al, 2007	0.63 (0.36, 1.12)	11/34	20/39	5.62
Jin et al, 2011	1.64 (1.02, 2.64)	38/58	12/30	4.77
**Subtotal (I−squared = 72.3%, p = 0.03)**	**1.00 (0.74, 1.34)**	**63/131**	**49/107**	**15.59**
**Gynecological cancer**				
Gupta et al, 2019	1.00 (0.07, 15.12)	1/25	1/25	0.30
**Subtotal**	**1.00 (0.07, 15.12)**	**1/25**	**1/25**	**0.30**
**Breast cancer**				
Falandry et al, 2009	1.45 (0.74, 2.84)	15/71	13/89	3.48
Dirix et al, 2008	1.05 (0.51, 2.15)	12/51	11/49	3.39
**Subtotal (I−squared = 0.0%, p = 0.52)**	**1.25 (0.77, 2.04)**	**27/122**	**24/138**	**6.87**
**Gastric cancer**				
Guo et al, 2019	1.31 (0.70, 2.45)	16/41	11/37	3.49
**Subtotal**	**1.31 (0.70, 2.45)**	**16/41**	**11/37**	**3.49**
**Head and neck cancer**				
Aghili et al, 2021	1.25 (1.06, 1.47)	56/60	44/59	13.39
**Subtotal**	**1.25 (1.06, 1.47)**	**56/60**	**44/59**	**13.39**
**Total**	**1.08 (0.96, 1.21)**	**376/1053**	**328/1008**	**100.00**

Overall heterogeneity (I−squared = 15.4%, p = 0.29)

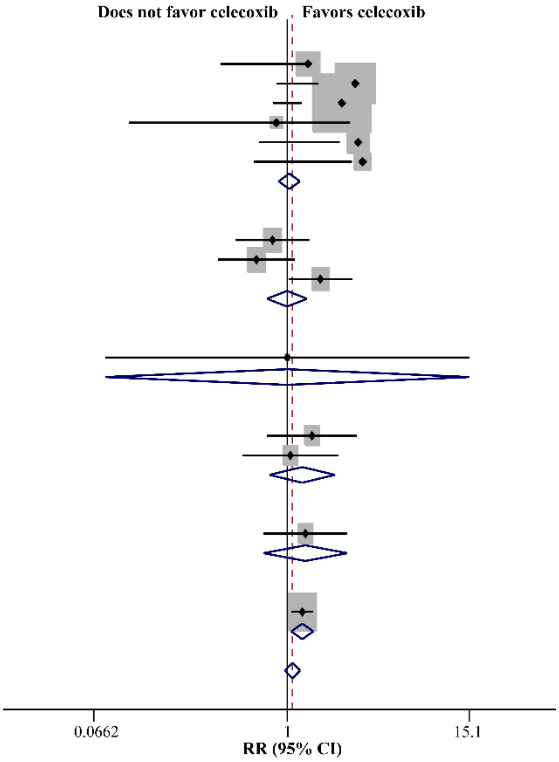

B

Source	RR (95% CI)	Celecoxib	Control	Weight %
**Lung cancer**				
Koch et al, 2011	1.05 (0.93, 1.19)	123/158	117/158	17.95
Reckamp et al, 2015	1.11 (0.82, 1.51)	34/53	30/52	4.65
Groen et al, 2011	1.02 (0.95, 1.10)	242/290	249/305	37.24
Lilenbaum et al, 2006	0.78 (0.46, 1.33)	16/51	20/50	3.10
Zhou et al, 2007	1.31 (1.00, 1.73)	28/32	22/33	3.32
**Subtotal (I−squared = 6.4%, p = 0.37)**	**1.04 (0.98, 1.11)**	**443/584**	**438/598**	**66.26**
**Colorectal cancer**				
Maiello et al, 2006	1.01 (0.79, 1.29)	30/39	29/38	4.51
Köhne et al, 2007	1.01 (0.85, 1.20)	30/34	34/39	4.86
Jin et al, 2011	1.39 (0.96, 2.01)	43/58	16/30	3.24
**Subtotal (I−squared = 35.2%, p = 0.21)**	**1.11 (0.95, 1.29)**	**103/131**	**79/107**	**12.60**
**Gynecological cancer**				
Reyners et al, 2012	0.90 (0.59, 1.36)	29/97	33/99	5.01
Gupta et al, 2019	1.11 (0.55, 2.26)	10/25	9/25	1.38
**Subtotal (I−squared = 0.0%, p = 0.61)**	**0.94 (0.66, 1.35)**	**39/122**	**42/124**	**6.39**
**Breast cancer**				
Falandry et al, 2009	1.08 (0.86, 1.34)	49/71	57/89	7.76
Dirix et al, 2008	0.96 (0.64, 1.44)	24/51	24/49	3.76
**Subtotal (I−squared = 0.0%, p = 0.62)**	**1.04 (0.85, 1.27)**	**73/122**	**81/138**	**11.52**
**Gastric cancer**				
Guo et al, 2019	1.22 (0.84, 1.76)	27/41	20/37	3.23
**Subtotal**	**1.22 (0.84, 1.76)**	**27/41**	**20/37**	**3.23**
**Total**	**1.05 (0.99, 1.11)**	**685/1000**	**660/1004**	**100.00**

Overall heterogeneity (I−squared = 0.0%, p = 0.76)

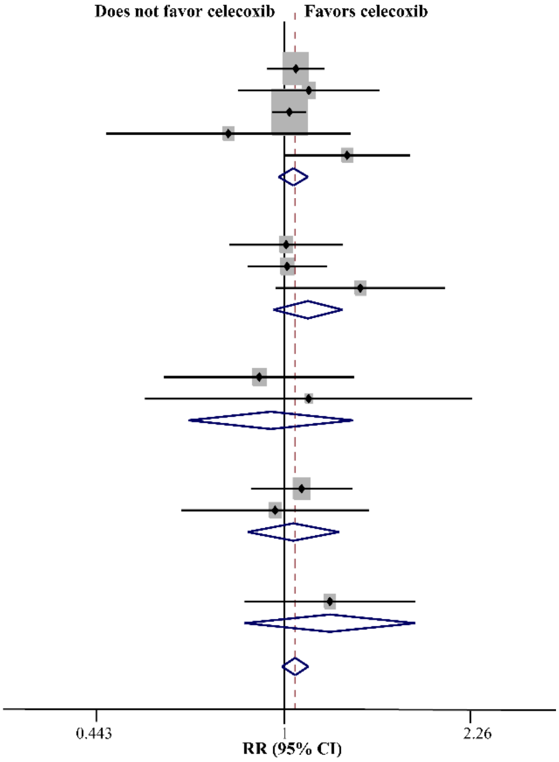

Figure S1. Local control efficacy of celecoxib-combined palliative therapy in different cancer types on objective response rate (ORR) (A) and disease control rate (DCR) (B). RR: relative risk; CI: confidence interval.

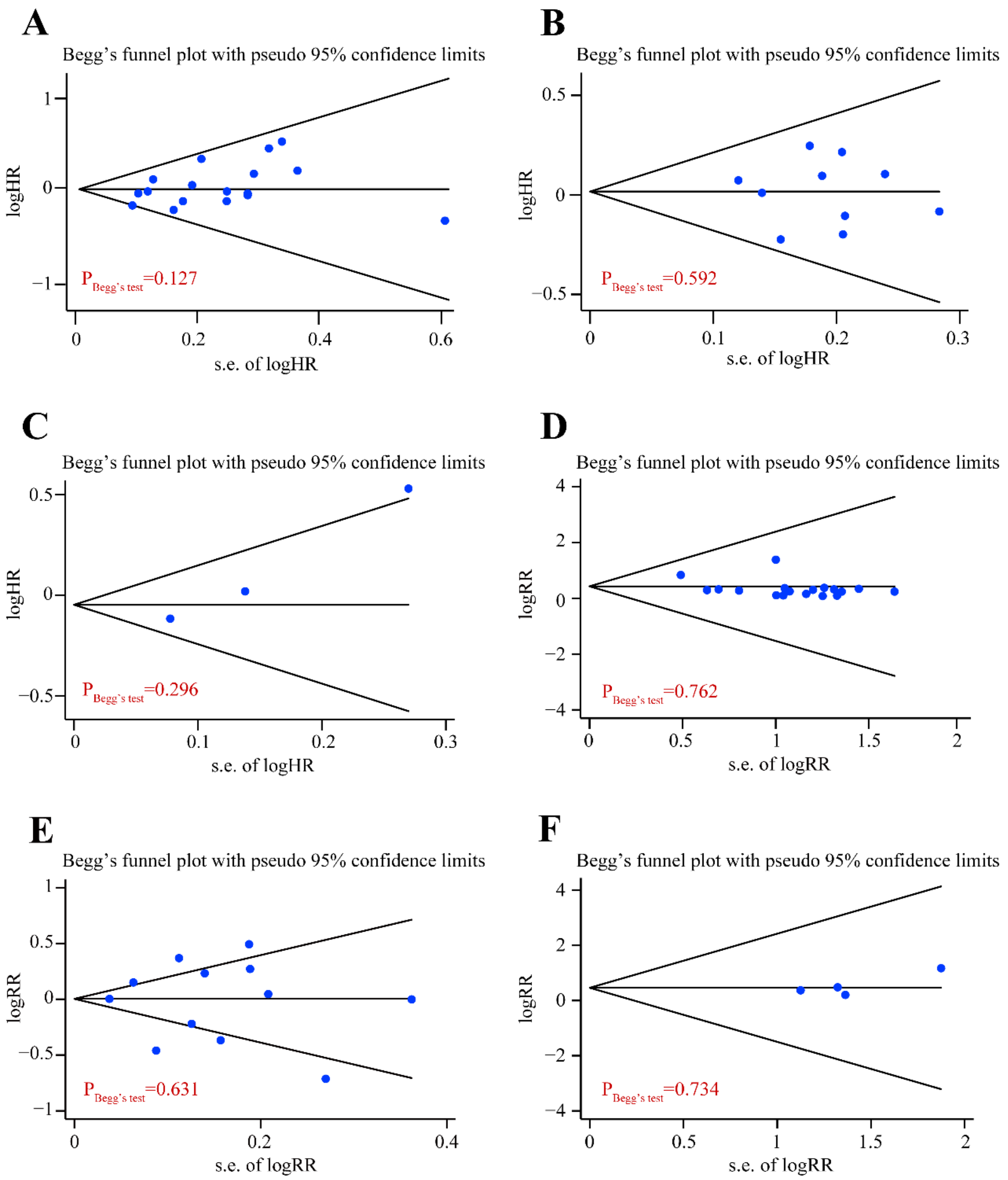

Figure S2: Begg's funnel plot of publication bias analysis in celecoxib-combined standard cancer therapy on overall survival (OS) (A), progression-free survival (PFS) (B), disease-free survival (DFS) (C), objective response rate (ORR) (D), disease control rate (DCR) (E), and pathological complete response (pCR) (F).

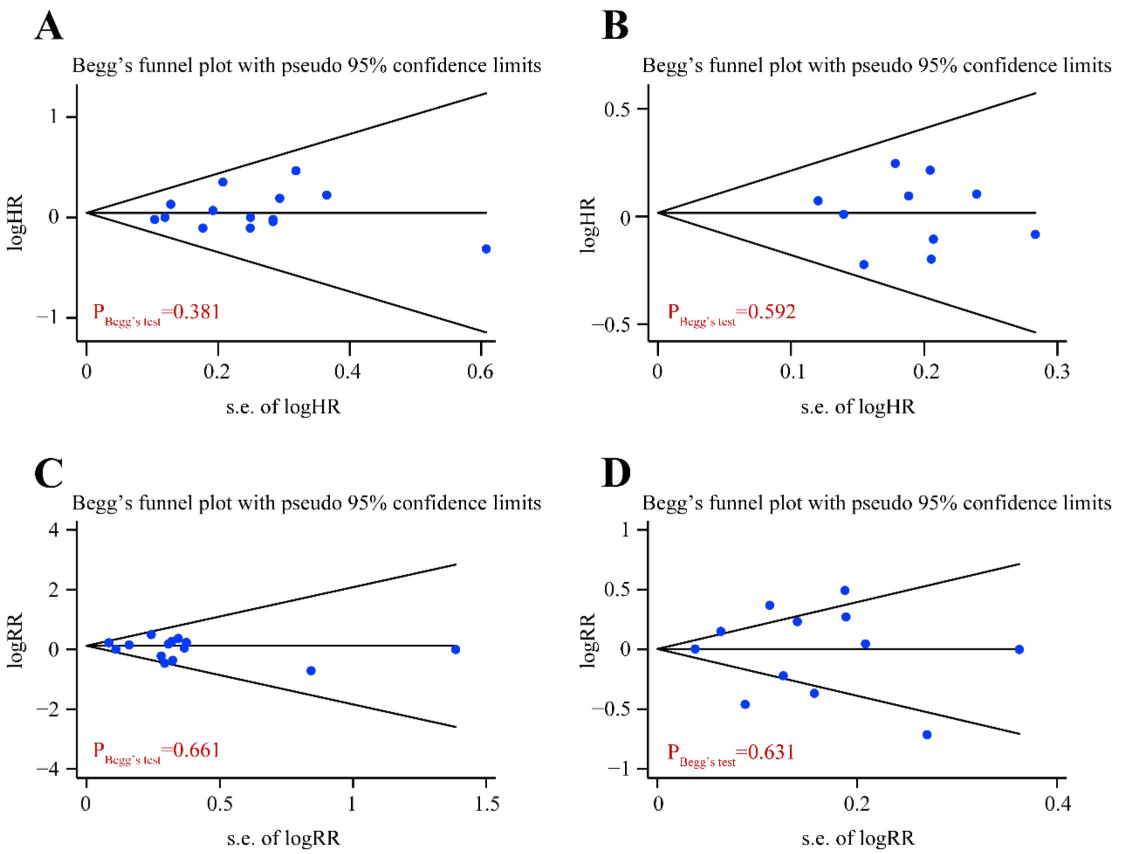

Figure S3: Begg's funnel plot of publication bias analysis in celecoxib-combined palliative therapy on overall survival (OS) (A), progression-free survival (PFS) (B), objective response rate (ORR) (C) and disease control rate (DCR) (D).

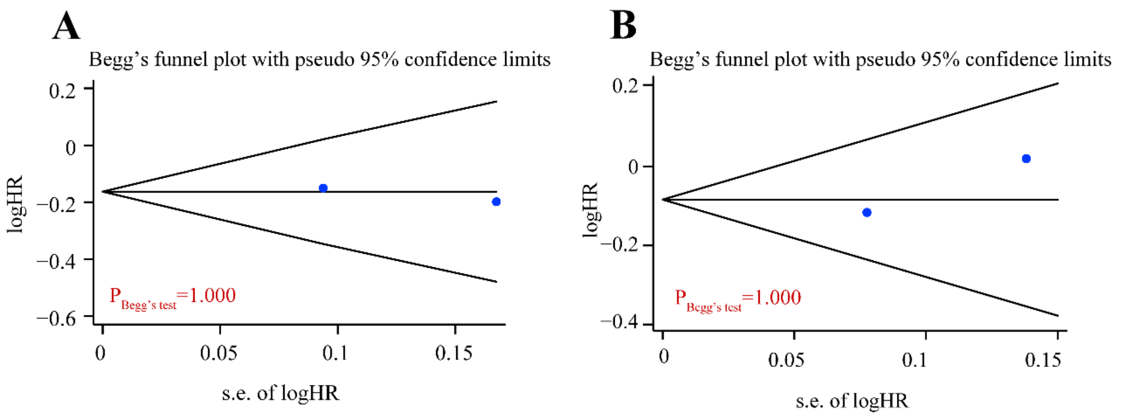

Figure S4: Begg's funnel plot of publication bias analysis in celecoxib-combined adjuvant therapy on overall survival (OS) (A) and disease-free survival (DFS) (B).

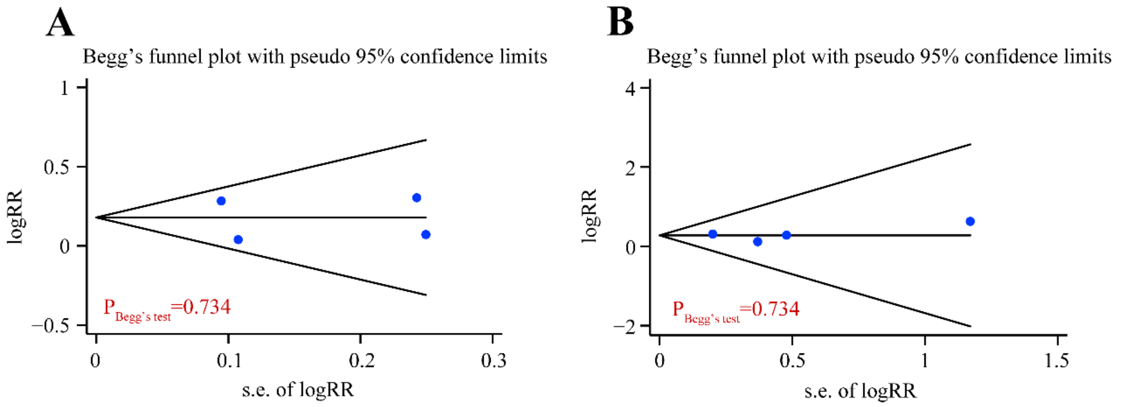

Figure S5: Begg's funnel plot of publication bias analysis in celecoxib-combined neoadjuvant therapy on objective response rate (ORR) (A) and pathological complete response (pCR) (B).

Table S1 Baseline characteristics and design variables of the included RCTs.

Author	Country &Year	Therapy Type	Sample Size	Cancer Type	Age (year): Median(range/Mean±SD)	Follow-up (Month)	Outcome Measured	Jadad Score
Edelman et al*	American 2017	1st palliative chemotherapy	422	Lung cancer	Median:64(range:36-89)	Median:31.0	OS, PFS, AEs	6
Reckamp et al	American 2015	≥1st palliative chemotherapy	109	Lung cancer	Median:63.5(range:41-80) for celecoxib; 65 (range:30-80) for control	Median:12.1	OS, PFS, Local Control, AEs	6
Koch et al*	Sweden 2011	1st palliative chemotherapy	319	Lung cancer	Median:66(range:38-85) for celecoxib; 65(37-85) for control	Median:36 (range:16-52)	OS, PFS, Local Control, AEs	7
Groen et al	Netherlands 2011	1st palliative chemotherapy	561	Lung cancer	Median:61(range:33-84)	NR	OS, PFS, Local Control, AEs	4
Lilenbaum et al	American 2006	2nd palliative chemotherapy	133	Lung cancer	Range:37-84	NR	OS, PFS, Local Control, AEs	2
Strasser-Weippl et al	American 2018	Adjuvant hormone therapy	1622	Breast cancer	Median:63.8	Median:49.2	OS, DFS	3
Maiello et al	Italy 2006	1st palliative chemotherapy	81	Colorectal cancer	Median:63(range:34-74) for celecoxib; 64(range:51-73) for control	Median:18 (range:10-32)	Local Control, AEs	2
Köhne et al	Germany 2008	1st palliative chemotherapy	85	Colorectal cancer	Median:64(range:42-78)	Median:14.6	OS, PFS, Local Control, AEs	3
Reyners et al	Netherlands 2012	1st palliative chemotherapy	202	Epithelial ovarian, fallopian tube, and primary peritoneal cancer	Median:59(range:30-83) for celecoxib; 61(range:27-85) for control	Median:32.3 (range:2-85)	OS, PFS, Local Control, AEs	2
Gupta et al	American 2019	≥1st palliative chemotherapy	52	Epithelial ovarian, fallopian tube, and primary peritoneal cancer	Median:61(range:27-80)	NR	OS, PFS, Local Control, AEs	4
Hamy et al*	France 2019	Neoadjuvant chemotherapy	220	Breast cancer	Median:47	Median:94.5	DFS, OS, AEs	4
Falandry et al	France 2009	Palliative hormone therapy	342	Breast cancer	Median:61(range:38-84) for celecoxib; 63(range:37-82) for control	Median:24	PFS, Local Control, AEs	6
Ahmadloo et al	Iran 2009	Neoadjuvant chemotherapy	50	Breast cancer	Median:42(range:24-70) for celecoxib;	NR	Local Control	5

					48(range:27-75) for control			
Guo et al	China 2019	Palliative chemotherapy	200	Gastric cancer	Median:54(range:28-70) for celecoxib; 56(range:32-69) for control	NR	Local Control, AEs	2
Bi et al	China 2019	1st palliative chemoradiation	100	Lung cancer	Median:60	Median:50 (range:32.2-73.6)	OS, PFS, AEs	6
Debucquoy et al	Belgium 2009	Neoadjuvant chemoradiation	80	Rectal cancer	Median:59(range:48-71) for celecoxib; 59(range:37-71) for control	Median:38.4 (range:8.1-50.4)	pCR, AEs	6
Edelman et al	American 2008	1st palliative chemotherapy	140	Lung cancer	NR	Median:29	OS, Local Control, AEs	2
Kelly et al	UK 2018	Palliative chemotherapy	570	Bladder cancer	Median:67(range:61-73)	Median:44 (range:36-57)	OS, DFS, AEs	7
JIN et al	China 2011	1st palliative chemotherapy	90	Colorectal cancer	NR	36	OS, Local Control, AEs	3
Rea et al	UK 2016	Noadjuvant hormone therapy	266	Breast cancer	NR	NR	Local Control	4
Mason et al	UK 2017	1st palliative hormone therapy	934	Prostate cancer	Median:65	Median:69	OS, AEs	4
Meyerhardt et al	American 2021	Adjuvant chemotherapy	2524	Colon cancer	Median:61.7(range:21.8-88.7) for celecoxib; 61(range:19.3-86.5) for control	Median:72	OS, DFS, AEs	6
Aghili et al	Iran 2021	1st palliative chemoradiation	122	Head and neck cancer	Mean±SD:56.46±14.65 for celecoxib; 55.08±12.45 for control	Median:30 (range:3-50)	OS, Local Control	6
Hu et al	China 2021	Neoadjuvant chemotherapy	34	Colorectal cancer	Median:45(range:23-69) for celecoxib; 53(range:31-69) for control	Median:14.9	pCR, AEs	4
Mohamma-Dianpanah et al	Iran 2011	1st palliative chemoradiation	50	Head and neck cancer	Median:43(range:18-69)	Median:26 (range:21-31)	Local Control, AEs	3
Chow et al	Japan 2004	Neoadjuvant chemotherapy	31	Breast cancer	Median:45.6	NR	Local Control, pCR	NR
Dirix et al	Belgium 2008	2nd palliative hormone therapy	111	Breast cancer	Median:56(range:32-82) for celecoxib; 56(range:28-79) for control	NR	Local Control, AEs	2

Chow et al	China 2008	Neoadjuvant hormone therapy	54	Breast cancer	Median:69(range:49-87) for celecoxib; 67(range:48-91) for control	NR	Local Control, AEs	3
Zhou et al	China 2007	1st palliative chemotherapy	65	Lung cancer	NR	NR	Local Control	1
Gharib et al	Egypt 2020	Adjuvant chemotherapy	86	Breast cancer	Median:48(range:28-60)	Median:59	DFS	2

*The studies have been merged with relevant data from other studies based on the same patient population. RCTs: Randomized clinical trials; UK: United Kingdom; NR: Not reported; SD: Standard deviation; OS: Overall survival; PFS: Progression-free survival; DFS: Disease-free survival; pCR: Pathological complete response; AEs: Adverse events.

Table S2. The results of subgroup analyses for the local control efficacy of celecoxib combined with palliative therapy.

	**RR**	**95%CI**	***P*-value**	**Heterogeneity (I^2, *P*-value)**
Objective response rate				
**Concomitant therapy strategies**				
Chemotherapy	1.04	0.90-1.19	0.61	11.2%,0.34
Hormone therapy	1.25	0.77-2.04	0.37	0.00%,0.52
**Therapy stages**				
1st line	1.09	0.96-1.23	0.17	37.6%,0.13
2nd line	0.90	0.47-1.73	0.75	0.00%,0.41
≥1st line	0.71	0.38-1.31	0.28	0.00%,0.80
**Sample size**				
<200	1.10	0.95-1.28	0.20	21.5%,0.22
≥200	1.05	0.88-1.26	0.56	0.00%,0.44
Disease control rate				
**Concomitant therapy strategies**				
Chemotherapy	1.05	0.99-1.11	0.12	0.00%,0.62
Hormone therapy	1.04	0.85-1.27	0.70	0.00%,0.62
**Therapy stages**				
1st line	1.05	0.99-1.11	0.13	1.40%,0.41
2nd line	0.88	0.64-1.22	0.44	0.00%,0.55
≥1st line	1.11	0.83-1.48	0.47	0.00%,1.00
**Sample size**				
<200	1.07	0.97-1.18	0.20	0.00%,0.67
≥200	1.03	0.97-1.10	0.34	0.00%,0.70

RR: relative risk; CI: confidence interval; I^2: I^2 showed the degree of heterogeneity.

Table S3. Meta-analysis of toxicities in cancer patients receiving celecoxib combined with palliative therapy.

Toxicity	RR	95%CI	*P*-value	Heterogeneity (I^2, *P*-value)
**Hematologic toxicities**				
Anemia	1.37	0.94-2.00	0.10	0.00%,0.81
Febrile neuropenia	1.04	0.74-1.46	0.82	0.00%,0.85
Neuropenia	1.11	0.96-1.27	0.16	0.00%,0.97
Thrombocytopenia	1.35	1.08-1.69	0.01	0.00%,0.69
Leucopenia	1.16	0.99-1.36	0.07	0.00%,0.57
Hemoglobin	1.33	0.84-2.10	0.22	18.40%,0.29
Lymphopenia	0.79	0.36-1.72	0.56	0.00%,0.57
Myelosuppression	0.51	0.18-1.46	0.21	0.00%,0.98
**Gastrointestinal toxicities**				
Overall	1.19	0.94-1.52	0.15	38.40%,0.08
Heartburn/dyspepsia	1.44	0.44-4.74	0.54	0.00%,0.62
Diarrhea	1.10	0.70-1.71	0.69	25.30%,0.24
Nausea	0.89	0.47-1.68	0.72	17.80%,0.30
Vomiting	1.47	0.62-3.47	0.38	16.00%,0.31
Nausea/Vomiting	1.05	0.66-1.67	0.85	19.60%,0.28
Constipation	3.00	0.31-28.59	0.34	0.00%,0.99
**Circulatory toxicities**				
Cardiac ischaemia/infarction	0.75	0.17-3.34	0.71	0.00%,0.57
Cerebrovascular ischaemia	3.66	0.61-22.08	0.16	0.00%,0.90
Thrombosis/thrombus/embolism	0.83	0.47-1.47	0.52	30.70%,0.22
**Musculoskeletal toxicities & neurotoxicity**				
Overall	0.92	0.61-1.41	0.71	0.00%,0.61
Neurotoxicity	0.36	0.11-1.25	0.11	0.00%,0.89
**Respiratory toxicities**				
Dyspnea	0.60	0.22-1.65	0.32	11.10%,0.34
Asthenia	0.46	0.07-3.16	0.43	0.00%,0.50
**Infection**				
Overall	1.25	0.63-2.49	0.52	30.80%,0.23
Esophageal infection	0.70	0.19-2.63	0.60	9.80%,0.29
**Pain**				
Overall	0.91	0.37-2.22	0.83	64.20%,0.06
Arthralgia	0.89	0.38-2.08	0.78	28.20%,0.24
Abdominal pain	1.37	0.46-4.06	0.57	0.00%,0.59
**Other toxicities**				
Fatigue	0.98	0.61-1.55	0.91	0.00%,0.88
Dehydration	0.73	0.27-1.96	0.54	0.00%,0.70
Anorexia	1.15	0.41-3.23	0.79	0.00%,0.67
Insomnia	1.09	0.47-2.53	0.84	32.90%,0.22
Rash	2.92	0.36-23.9	0.32	64.70%,0.04
Mucositis	0.77	0.55-1.08	0.13	38.30%,0.18
Allergic reaction	1.07	0.52-2.19	0.86	0.00%,0.62

RR: relative risk; CI: confidence interval; I^2: I^2 showed the degree of heterogeneity.

Table S4. The analysis results of publication bias in celecoxib combined with standard cancer therapy.

	Begg's test	Egger's test
**Overall analyses**		
Overall survival (OS)	0.127	0.089
Progression-free survival (PFS)	0.592	0.907
Disease-free survival (DFS)	0.296	0.106
Objective response rate (ORR)	0.762	0.059
Disease control rate (DCR)	0.631	0.903
Pathological complete response (pCR)	0.734	0.890
**Palliative therapy**		
Overall survival (OS)	0.381	0.518
Progression-free survival (PFS)	0.592	0.586
Objective response rate (ORR)	0.661	0.311
Disease control rate (DCR)	0.631	0.903
**Adjuvant therapy**		
Overall survival (OS)	1.000	/
Disease-free survival (DFS)	1.000	/
**Neoadjuvant therapy**		
Objective response rate (ORR)	0.734	0.949
Pathological complete response (pCR)	0.734	0.192

"/": not applicable due to limited number of studies.

PRISMA checklist

Section/topic	#	Checklist item	Reported on page #
**TITLE**			
Title	1	Identify the report as a systematic review, meta-analysis, or both.	1
**ABSTRACT**			
Structured summary	2	Provide a structured summary including, as applicable: background; objectives; data sources; study eligibility criteria, participants, and interventions; study appraisal and synthesis methods; results; limitations; conclusions and implications of key findings; systematic review registration number.	1
**INTRODUCTION**			
Rationale	3	Describe the rationale for the review in the context of what is already known.	1-2
Objectives	4	Provide an explicit statement of questions being addressed with reference to participants, interventions, comparisons, outcomes, and study design (PICOS).	3
**METHODS**			
Protocol and registration	5	Indicate if a review protocol exists, if and where it can be accessed (e.g., Web address), and, if available, provide registration information including registration number.	4
Eligibility criteria	6	Specify study characteristics (e.g., PICOS, length of follow-up) and report characteristics (e.g., years considered, language, publication status) used as criteria for eligibility, giving rationale.	3
Information sources	7	Describe all information sources (e.g., databases with dates of coverage, contact with study authors to identify additional studies) in the search and date last searched.	2
Search	8	Present full electronic search strategy for at least one database, including any limits used, such that it could be repeated.	3
Study selection	9	State the process for selecting studies (i.e., screening, eligibility, included in systematic review, and, if applicable, included in the meta-analysis).	3
Data collection process	10	Describe method of data extraction from reports (e.g., piloted forms, independently, in duplicate) and any processes for obtaining and confirming data from investigators.	3
Data items	11	List and define all variables for which data were sought (e.g., PICOS, funding sources) and any assumptions and simplifications made.	3, 15
Risk of bias in individual studies	12	Describe methods used for assessing risk of bias of individual studies (including specification of whether this was done at the study or outcome level), and how this information is to be used in any data synthesis.	4

Summary measures	13	State the principal summary measures (e.g., risk ratio, difference in means).	3-4
Synthesis of results	14	Describe the methods of handling data and combining results of studies, if done, including measures of consistency (e.g., I^2) for each meta-analysis.	3-4
Risk of bias across studies	15	Specify any assessment of risk of bias that may affect the cumulative evidence (e.g., publication bias, selective reporting within studies).	3-4
Additional analyses	16	Describe methods of additional analyses (e.g., sensitivity or subgroup analyses, meta-regression), if done, indicating which were pre-specified.	3-4
**RESULTS**			
Study selection	17	Give numbers of studies screened, assessed for eligibility, and included in the review, with reasons for exclusions at each stage, ideally with a flow diagram.	4
Study characteristics	18	For each study, present characteristics for which data were extracted (e.g., study size, PICOS, follow-up period) and provide the citations.	4
Risk of bias within studies	19	Present data on risk of bias of each study and, if available, any outcome level assessment (see item 12).	12
Results of individual studies	20	For all outcomes considered (benefits or harms), present, for each study: (a) simple summary data for each intervention group (b) effect estimates and confidence intervals, ideally with a forest plot.	5-12
Synthesis of results	21	Present results of each meta-analysis done, including confidence intervals and measures of consistency.	5-12
Risk of bias across studies	22	Present results of any assessment of risk of bias across studies (see Item 15).	5-12
Additional analysis	23	Give results of additional analyses, if done (e.g., sensitivity or subgroup analyses, meta-regression [see Item 16]).	5-12
**DISCUSSION**			
Summary of evidence	24	Summarize the main findings including the strength of evidence for each main outcome; consider their relevance to key groups (e.g., healthcare providers, users, and policy makers).	13-14
Limitations	25	Discuss limitations at study and outcome level (e.g., risk of bias), and at review-level (e.g., incomplete retrieval of identified research, reporting bias).	14
Conclusions	26	Provide a general interpretation of the results in the context of other evidence, and implications for future research.	14
**FUNDING**			
Funding	27	Describe sources of funding for the systematic review and other support (e.g., supply of data); role of funders for the systematic review.	15
